# Molecular docking and simulation analysis of nimbolide with poly-galacturonase from *Aspergillus niger*: Managing black mold disease for *Allium cepa*

**DOI:** 10.6026/973206300211050

**Published:** 2025-05-31

**Authors:** Pranshu Dangwal, Saransh Juyal, Arun Bhatt, Mamta Baunthiyal, Dev Bukhsh Singh

**Affiliations:** 1 Department of Biotechnology, Govind Ballabh Pant Institute of Engineering & Technology, Pauri Garhwal, Uttarakhand-246194, India; 2 Department of Biotechnology, Siddharth University, Kapilvastu, Siddharth Nagar, India

**Keywords:** *Allium cepa*, phytoalexins, nimbolide, polygalacturonase, molecular modelling, molecular docking

## Abstract

Black mold disease is a major post-harvest issue in *Allium cepa* caused by *Aspergillus niger*. Therefore, it is of interest to describe the molecular docking and simulation analysis of poly-galacturonase protein from *Aspergillus niger* that is involved in disease progression as a promising molecular target for the identification of novel fungicides. Hence, we used I-TASSER to model the protein and docked it with the naturally occurring phytoalexins, which included nimbolide, nimbolin, Azadiradione, Quercetin and Azadirone. We show that nimbolide has the greatest affinity towards poly-galacturonase as compared to other phytoalexins binding with residues Gln205, Gln261, Tyr262 having four hydrogen bonds and -8.0 kcal/mol binding energy. Further, molecular dynamics simulation of protein and docked nimbolide-polyglacturonase complex was carried out to validate the stability of the system at the atomic level. Based on the study, the molecule shows potential for inhibiting pathogenic proteins, making it a promising candidate for further validation under laboratory and field conditions to ensure food and nutritional security.

## Background:

*Allium cepa*, commonly known as onion, is a major vegetable crop grown all over the world. Onion is called "Pyaz" or "Kanda" in Hindi, belongs to the family Alliaceae and is used for cooking or used in the form of salad. Onion has a number of medicinal benefits [1]. Black mold rooted by *Aspergillus niger* van Tieghem (An) onion acts as a restraining aspect in onion -yield globally [2]. The presence of *Aspergillus niger* as a soil saprophyte has been reported, whenever they find wounded tissues, it attacks/infects onion bulbs in the field or storage by secreting a different enzyme or toxin have reported the relationship of *Aspergillus niger* with seeds of onion-shaped in warm (wasteland) climates and how the onion seedling get infected through the soil and already infected seeds, which can affect 30 to 80% damage of bulbs of onions [3]. The handling of seeds with different biocides like leaf extract of plants in place of different fungicides has been reported to be safe. In several crops including onion, it has been reported that biocides help in increasing the germination of seeds and by reducing vigor index, the initial- and later-coming-out death [4, 5]. Plants produce several secondary metabolites as a defence mechanism against pests and pathogens. These low molecular mass metabolites which show antimicrobial properties are collectively known as phytoalexins [6]. Phytoalexins are considered a molecular marker of disease resistance that shows natural action towards 173 varieties of pathogens [7, 8] they are a miscellaneous compound [9]. The conception of phytoalexin was introduced many years ago based on a report that potato (Solanum tuberosum) tuber tissue contaminated with an irreconcilable species of *Phytophthora* (*Phytophthora infestans*) develops induced resistance to a well-suited race of *P.infestans*. To understand the accurate instrument through which phytoalexin exerts its toxicity is at rest unidentified, however it has been shown the powerfully inhibit conidial germination and germ tube elongation and also damage the cubicle crust of plant pathogens. Phytoalexins are well thought-out as necessary compounds for plant- resistance against pathogens however they are yet to be characterized in most of the plant species [10]. Therefore, it is of interest to describe the molecular docking and simulation analysis of poly-galacturonase protein from *Aspergillus niger* that is involved in disease progression as a promising molecular target for the identification of novel fungicides.

## Materials and Methods:

## Series repossession and analysis of physico-chemical properties:

The protein sequence of the pathogen, polygalacturonase (529bp), was obtained from the "National Centre for Biotechnology Information (https://www.ncbi.nlm.nih.gov) database. A comprehensive analysis of its physico-chemical properties was conducted, which included determining various parameters such as molecular weight (MW), amino acid composition, theoretical isoelectric point (pI), aliphatic index (AI) and extinction coefficient, grand average of hydropathicity (GRAVY), estimated half-life and instability index. For the analysis of the entire key chain of the target protein, we utilized ProtParam (http://web.expasy.org/protparam/), a tool for understanding and analysing proteins, available on the ExPASy server.

## Protein secondary and tertiary structure estimation:

Secondary structural analysis of protein sequence involves assigning various regions that are expected to associate with secondary structures such as alpha helices, beta strands, or turns. Protein structure prediction server PSIPRED [11, 12] and a Self-optimised prediction method with alignment (SOPMA) were applied to predict the less important structures of the target proteins. Iterative threading assembly refinement (I-TASSER) [13, 14] was used to predict the 3-D structures of the target proteins. The tertiary structural examination is performed to predict the arrangement of the secondary structure, along with its side chains, into 3-D. The tertiary structure of the protein mostly decides its biological function. I-TASSER mechanically develops tall quality 3-D structure of the protein molecule from the amino acid sequence and ultimately uses this structure and amino acid sequences to predict the biological function of that protein molecule. It executes numerous threading algorithms and iterative formation-assembly simulations to discover the best possible sub-fragments inside the folder of structures or the client-specific composition [15]. Cello, prediction tools were applied to determine the sub-cellular localization of the queries of the protein.

## Structure evaluation:

Various structures anticipated by I-TASSER were authenticated by PROCHECK, as single-minded by Ramachandran plot information. I-TASSER generated the top four protein models; the model with the highest C-value was selected for further studies.

## Ligand preparation:

The structure of different phytoalexins, *viz.* Nimbolide, nimbolin, azadiradione, quercetin, azadirone, oleuropein was retrieved from PubChem database of "National Centre for Biotechnology Information" (http://pubchem.ncbi.nlm.nih.gov). The three-dimensional coordinates of ligand molecules were generated by Marvin Sketch (http://www.chemaxon.com /products/marvin/marvinsketch/) software and saved in PDB file format. The protein data bank (PDB) file was then converted into pdbqt format using Autodock tools, which can be used for docking [16].

## Docking:

The studies of molecular docking were done by AutoDock vina by means of the prepared 3D construction of different phytoalexins with polygalacturonase as the molecular target. For every ligand, we chose all conformers based on their optimal interaction, considering their docking energy and the count of hydrogen bonds. The examination and illustration of the protein-ligand interaction were accomplished using Ligplot [17].

## Molecular dynamics simulation:

The MDS study was executed using GROMACS 4.6.5 [18]. A two-system approach was formed and engaged for 10 ns time period reproduction studies, the first system is to predict the stability of a 3-D model of protein and another for protein-ligand composite. Both systems were immersed in a cubic container using a basic point charge concept. The ligand topology was created via the ProDRG programme. The protein topology was built via the GROMOS 9653a6 force field [19]. A total of 16 sodium ions were introduced to the systems in order to achieve neutralisation. The systems were subjected to a consistently intense energy minimization process in order to achieve the highest power output below 1000 kj/mol/nm and eliminate any conflicting interactions. The atom Mesh Ewald method was utilised to quantify electrostatic interactions. Hydrogen bond lengths were restricted using the LINCS technique. The reproduction was programmed to occur at a pace of 2 femtoseconds. A short-range non-bonded interaction was anticipated, with a predicted cut-off distance of 10 Å. Extended-range electrostatics was computed in the PME system with a 1.6 Å Fourier grid spacing. The Shake algorithm was used to predefine all the bonds, including hydrogen bonds. Simulations for NVT and NPT were executed for duration of 1 n. Subsequently, both systems underwent Molecular Dynamics Simulations (MDS) for 10 ns. Root Mean Square Fluctuation (RMSF) and Deviation (RMSD) were analysed. Image molecular dynamics and Chimaera 1.10.2 were used for the trajectory analysis [20]. The resulting plots were generated and visualized using the Origin tool.

## Results and Discussion:

The protein was analysed by means of the ProtParam server. A polygalacturonase protein has 58.47 kDa as its theoretical pI value and 529 amino acids as its molecular weight. The residue composition of this enzyme was found as Ala (6%), Arg (3.4%), Asn (8.7%), Asp (7.6%), Cys (1.9%), Gln (3.8%), Glu (3.6%), Gly (9.1%), His (2.5%), 33 (6.2%), Leu (6.2%), Lys (3.2%), Met (2.3%), Phe (3.4%), Pro (4.5%), Ser (6.6%), Thr (6.4%), Trp (2.6%), Tyr (4.2%) and Val (7.8%). The total count of negatively charged residues (comprising Aspartic Acid and Glutamic Acid) and positively charged residues (consisting of Arginine and Lysine) was determined 59 and 35, correspondingly. A total number of atoms present in the target protein were 8038, with the chemical formula C2585H3920N706O805S22. Indicating the light absorption capacity, the extinction coefficient is computed at 280 nm and expressed in M-1 cm-1. Estimate half-life when M (Met) is considered as the N-terminal of the sequence. The projected half-life of the polygalacturonase protein was predicted 30 hours for mammalian reticulocytes in vitro model. Instability index of the polygalacturonase protein was found 37.97 that indicate a stable form of protein. Aliphatic index (AI) of a protein has a direct relation with the volume engaged by the surface chains of amino acids (alanine, leucine, valine and isoleucine).

Thermal stability of the globular proteins increases with an increase in AI value. Aimed at protein, the aliphatic- index was computed to be (73.18). The GRAVY index for this protein was -0.331, which indicates its better transportation through the water (medium) solvent. SOPMA, a software tool, was used for Secondary structure analysis of protein. Polygalacturonase protein consists of 18.90 % alpha-helices, 39.51% haphazard coils, 34.40%, extended beta strands and 7.18% beta turns (Figure 1 see PDF). The sub-cellular localization by Cello indicates that this is an extracellular protein. The 3-D structure of Polygalacturonase was modeled using I-TASSER, which is based on the threading approach (Figure 2 see PDF). The quality of the tertiary model was assessed with the help of the PROCHECK tool and the related Ramachandran plot result indicates that 38.5% of residues belong to favoured regions while 42.2% of residues reside in allowed regions. The liberally allowed region contains 12.3% residues, whereas 7% residues are prohibited or outlier regions (Figure 3 see PDF). The structure of polygalacturonase was constructed by I-TASSER. This structure was analyzed by AutoDock tool and converted into. pdbqt file format after the addition of polar hydrogen. I- TASSER also determined the binding site residues of the protein, Gln205, Gln261, Tyr262, Asn270, Ile271, Val273 and Asn320 were found to be present at active site. The grid map centred at the active site pocket of protein (www.scfbio.iitd.res.in) lies in the centre x 66.361; centre y 66.444, centre z 68.583 with size 46, 44, 42. Structures of phytoalexins were downloaded from the PubChem database. 3-dimensional coordinates of phytoalexins were organized by using Marvin sketch in .pdb format and these files were used to prepare. pdbqt files by autodock for molecular docking studies. The studies of molecular docking were made by AutoDock vina by means of the prepared 3-D structure of different phytoalexins, *viz.* Nimbolide, nimbolin, Azadiradione, Quercetin and Azadirone with polygalacturonase as molecular target whereas the visualization and analysis of protein ligand complex was done using ligplot. Nimbolide, nimbolin, Azadiradione, Quercetin and Azadirone docked with polygalacturonase with docking energy -8.0, -8.0,-7.8,-7.6,-7.4 kcal/mol, respectively. Hydrogen bonds between phytoalexins and amino acid residues of pathogenic protein are Figure 4 - (see PDF). Table 1 shows that, the binding-free energy of all phytoalexins with the target protein obtained through molecular docking studies. The stability of the projected protein model was assessed during a 10 ns MD simulation and the binding mode of the protein-ligand complex was also analyzed using a 10 ns MD simulation. MDS applying solvent, pressure and set temperature predicted the mechanism of precise binding of the complex. We computed the root mean square volatility and deviation from the trajectory [5]. RMSD serves as an indicator of system stability. The RMSD of both the protein and the protein-ligand complex was observed to increase between 1 and 4 ns, indicating that both structures remained stable as they dissolved in the cubic box solution and any internal repulsion was eliminated over time. After 5 ns, both systems reached equilibrium and maintained a steady trajectory for analysis. The protein and protein-ligand complex exhibited average RMSDs of 0.43 nm and 0.38 nm, respectively (Figure 5 see PDF). The RMSD values suggest that the protein-ligand complex was more stable compared to the protein alone. We computed RMSF values to compare the flexibility of every amino acid residue in the complex and the protein. RMSF gives light on the structural differences of every residue. Lower RMSF values indicate well-structured regions, while higher values suggest more flexible or disordered areas, such as loops or lethal domains. In the study, it was calculated the value of RMSF for the 10 ns. The peak of RMSF protein- ligand complex was to some extent higher than protein; however the average RMSF value was 0.15 nm and 0.14 nm for protein and protein-ligand complex (Figure 6 see PDF). The black and red line represents poly-galacturonase and poly-galacturonase-nimbolide, respectively. The complex projected less variation as comparison to the protein. Utilising Rg, the conformational variations and hardness of the protein-ligand complexes and the apo-protein were determined. The Rg value was determined for all the complexes with the apo-protein using the 10 ns trajectories. For the apo-protein and protein-ligand complex, the average Rg values were determined to be 2.38 and 2.33 nm, respectively (Figure 7 see PDF). Protein-ligand complex showed lesser Rg value in comparison to apo-protein. The result suggested that protein ligand complex is more stable than the apo-protein. Protein-ligand complexes are stabilised by many interactions, including hydrophobic, electrostatic and hydrogen bonds. Highly definite and transitory interactions, hydrogen bonds, are a crucial component of protein-ligand stabilisation. The many hydrogen bonds vs. time are explained in (Figure 8 see PDF). For the protein-ligand complex, the hydrogen bonds were counted normally between 0 and 1. It shows how the ligand keeps creating hydrogen bonds right up until the simulation is over. Phytoalexins play a significant role in plant fighting in opposition to plant pathogens, not only in dicot species but in monocots as well. It has been lately depicted that assault of maize stem by Rhizopus microspores and Collect otrichumgraminicola induces the gathering of 6 ent- Lausanne pertaining to diterpenoids, commonly termed kauralexins, which hamper the expansion of the pathogens. The outcome of the current study noticeably shows, phytoalexin nimbolide can perform a direct molecule in the protection against fungal diseases. Nimbolide is the tiny hydrophobic molecule which can do cross cell membranes because of its perfect logP value and lower molecular weight, which can maintain diffusion of the hydrophobic molecule in the course of the membrane. It has been ascertained that nimbolide have shown the highest affinity towards pathogenic proteins of *Aspergillus niger*. Therefore, nimbolide could be positive for safeguarding the *Allium cepa* in opposition to fungal diseases together including black mold (Figure 9 see PDF).

## Conclusion:

The phytoalexin nimbolide shows strong binding affinity with the pathogenic polygalacturonase protein of *Aspergillus niger*, suggesting its potential as a lead molecule against black mold disease in *Allium cepa*. Structural modifications of nimbolide were proposed to design potent, eco-friendly antifungal agents as alternatives to harmful synthetic fungicides. These findings provide a foundation for future wet lab validation and the development of sustainable plant protection strategies.

## Figures and Tables

**Table 1:** Binding- free energy of all phytoalexins with the target protein obtained through molecular docking studies.

**S No.**	**Name of compound**	**Free Binding energy (Kcal/mol)**	**No. of Hydrogen bond**	**Interacting amino acid residue through Hbond**
1	nimbolide	-8	4	Gln205, Gln261, Tyr262
2	nimbolin	-8	3	Lys235, Ser258
3	Azadiradione	-7.8	3	Asn176, Asn179, Tyr262
4	Quercetin	-7.6	7	Gln205, Asp229, Asp230, Ser258, Tyr290, lys292
5	Azadirone	-7.4	3	Ser100, Gln205, Lys235
6	Oleuropein	-7.4	13	Gln205, Asn206, Asp229, lys292, Asp230,lys235, Gln261, Tyr262
7	Nimbin	-7.3	7	Trp173, Asn206, Ser258, Gln261, Tyr262
8	Salannin	-7.3	2	Arg237, Gln261
9	Nimbinene	-7.1	3	Asn206, Lys235, Ser258
10	Desacetylnimb in	-7	3	Trp155, Asn176, Tyr262
11	Beta sitasterol	-6.9	2	Gln205, Asp229
12	caryophyllene	-6.1	-	No significant interaction
13	Caffeic acid	-6	7	Gln64, lys86, Thr88, Asn134, Asp169, Gln471
14	Carvacrol	-5.9	7	Ile45, Met46, Phe49, Ala48, Phe49, Glu50, Glu51, Cys52, Gly53
15	Protocatechoic acid	-5.8	5	Val204, Gln205, Gly228, Asn252, Tyr281
16	4-hydroxy benzoic acid	-5.6	4	Gly228, Asn252, Tyr281, Asp284
17	Syringic acid	-5.6	6	Trp173, Asp208, Ser258, lys292
18	Vanillic acid	-5.5	3	Trp203, val204, Tyr281
19	Tsibulin2	-5.4	2	Asn252, Tyr281
20	linalool	-5.1	2	Trp203, val204
21	Pyrocatechol	-4.8	5	Asn134, Lys168, Asp169, Gln471
22	Allicin	-4.2	1	Asn252

## References

[1] Oyawoye O.M (2022). Bull Natl Res Cent..

[2] Pudake S (2019). International Journal of Chemical Studies..

[3] Murray J (2023). SPIE 12383 Imaging, Manipulation, and Analysis of Biomolecules, Cells, and Tissues.

[4] Zhang Y (2008). BMC Bioinformatics..

[5] Lipinski C.A (2001). Adv Drug Deliv Rev..

[6] Nakamura M (2019). J Gen Plant Pathol..

[7] Schüttelkopf A.W (2004). Acta Crystallogr D Biol Crystallogr..

[8] Schmelz E.A (2011). Proc Natl Acad Sci..

[9] Geourjon C (1995). Comput Appl Biosci..

[10] Bizuneh G.K (2021). Adv Tradit Med..

[11] Zhu S (2019). IOP Publishing..

[12] Patel P (2016). Int J Adv Res Comput Commun Eng..

[13] Huffaker A (2011). Plant Physiol..

[14] Jani J (2021). Advances in Bioinformatics..

[15] Yang J (2015). Nucleic Acids Res..

[16] Yakobi S (2024). Chemistry Select..

[17] Trott O (2010). J Comput Chem..

[18] Taghvaei S (2022). PPAR Res..

[19] Oostenbrink C (2004). J Comput Chem..

[20] Badar M.S (2022). Transdisciplinarity. Integrated Science.

